# Ionizing Radiation-Induced Adaptive Response in Fibroblasts under Both Monolayer and 3-Dimensional Conditions

**DOI:** 10.1371/journal.pone.0121289

**Published:** 2015-03-25

**Authors:** Yinlong Zhao, Rui Zhong, Liguang Sun, Jie Jia, Shumei Ma, Xiaodong Liu

**Affiliations:** 1 Key Laboratory of Radiobiology (Ministry of Health), School of Public Health, Jilin University, Changchun, China; 2 Dept. Nuclear Medicine, 2nd Hospital Jilin University, Changchun, China; 3 Dept. Translational Medicine, 1st Hospital Jilin University, Changchun, China; 4 Dept. Ultrasound, China-Japan Union Hospital, Changchun, China; University of California Davis, UNITED STATES

## Abstract

To observe the adaptive response (AR) induced by ionizing radiation in human fibroblasts under monolayer and 3-dimensional (3-D) condition. Three kinds of fibroblasts were cultured under both monolayer and 3-D condition. Immunofluorescent staining was used to detect the γ-H2AX foci and the morphological texture. Trypan blue staining was used to detect the cell death. Western blot was used to detect the expressions of γ-H2AX, p53 and CDKN1A/p21 (p21). We found that DNA damage increased in a dose-dependent and time-dependent manner after high doses of radiation. When cells were pretreated with a priming low dose of radiation followed by high dose radiation, DNA damage was attenuated under both monolayer and 3-D condition, and the adaptive response (AR) was induced. Additionally, the morphology of cells under monolayer and 3-D conditions were different, and radiation also induced AR according to morphological texture analysis. Priming low dose radiation induced AR both under monolayer and 3-D condition. Interestingly, 3-D microenvironment made cells more sensitive to radiation. The expression of p53 and p21 was changed and indicated that they might participate in the regulation of AR.

## Introduction

Accumulating evidences have shown that the biological effects of low-dose radiation are different from that of high dose radiation. Adaptive response (AR) is a form of cellular response that could be induced by low doses of radiation (priming dose, D1) followed by higher dose of radiation (challenging dose, D2), the chromosome aberrations that D2 caused will be attenuated by the pretreatment of D1 [1]. Adaptive response involves the activation of numerous signaling pathways [2–5]. Growing evidences have shown that the cell responses to ionizing radiation through genes associated with DNA repair, stress response, cell cycle control and apoptosis. TP-53 plays important roles in control of the low-dose radioadaptive response [2, 6, 7]. The microarray analysis performed by Lanza et al has shown that 111 genes are modulated at different doses of irradiation. And the cells response to low doses by the upregulation of the protein kinase C through p38 MAP kinase led to the activation of P53[3]. Recent studies have demonstrated that poly-ADP-Ribose Polymerase-1 (PARP-1) is also involved in AR induced by low dose of ionizing radiation by interfering in the cell cycle and apoptosis [8]. Several possible processes might involve in the protective effect of AR, including antioxidant defense mechanisms, DNA repair activation [9]. Olivieri et al [10] first found fewer chromatid aberrations when human lymphocytes were grown in low concentrations of radioactive thymidine and then exposed to high dose of radiation, as compared with exposure to high dose of radiation alone. AR was also reported by the pretreatment of human lymphocytes with non-ionizing radiofrequency fields followed by 1.0 Gy or 1.5 Gy X ray [11].

DNA double-strand breaks (DSBs) are generally accepted to be the most significant biological lesion associated with the ionizing radiation-related cancer and hereditary disease. H2AX is one of the highly conserved histone proteins that package the DNA into chromatin. When cells are irradiated, H2AX would be posphorylated (γ-H2AX) and the foci of γ-H2AX are detectable. Therefore, γ-H2AX has been used as an effective marker for DSBs [12–14].

3-D cell culture systems are essential tools because they more closely mimic natural tissues and organs than cells grown in 2D. The 3-D cell culture technique has been used in neurodegenerative disorders and drug discovery studies and serves as a precise human neural cell model [15–17]. In 3-D cell culture, the extracellular matrix is the natural material to which cells are attached and provides important biological instructions to the cells. 3-D cell culture environment more accurately simulates normal cellular processes including morphology, proliferation, differentiation and migration [16, 17]. Some studies indicated that under 3-D culture system ionizing radiation could induce senescence-like effects on fibroblasts and contribute to breast carcinogenesis by perturbing the tissue microenvironment that leads to dysregulated cell-cell and cell-matrix interactions [18]. However, no studies are done to examine the AR under 3-D condition. Here, we studied the radiation-induced AR under different culture conditions. Our results showed that priming radiation could induce AR on fibroblasts both under monolayer and 3-D conditions. The fibroblasts are much more sensitive to radiation when cultured at 3-D conditions.

## Materials and Methods

### Cell lines and reagents

Human fibroblasts RMP-4 cells were obtained from Brigham and Women's Hospital Boston, IMR-90 and mouse fibroblast MEF cell lines were purchased from ATCC. These cells were used within 10 passages in this study. For immunofluorescence staining, 3000 2-D cultured fibroblasts were seeded on sterile glass cover slips in Petri dish, and 500 3-D cultured cells were seeded in sterile 8-well chamber slides. Rat tail collagen I was purchased from BD Biosciences (San Diego, CA). The following primary antibodies were used: pholloidin was purchased from Chemicon (Temecula, CA). beta-actin mAb and propidium iodide were purchased from Sigma; p53 was purchased from Oncogene; p21waf1 was purchased from Calbiochem; γ-H2AX was purchased from Sigma. The following secondary antibodies were used: Alexa Fluor-488–labeled goat anti-rat and anti-mouse immunoglobulin from Molecular Probes (Eugene, OR).

#### Monolayer Cell culture

Cells were cultured in 95% air and 5% CO_2_ at 37°C in DMEM medium supplemented with 15% FBS, 1x non-essential amino acid, 1x HEPES, 0.0005% 2-mercaptoethanol and 1x antibiotics. For passaging, cells were washed with Ca^2+^/Mg^2+^-free PBS, then incubate with trypsin-EDTA mixture (0.05% tripsin, 0.53 mM EDTA) for 3–15 min at 37°C incubator. Cells were dislodged by tapping, neutralized with 2% calf serum in medium. After centrifugation cells were counted and seeded in plates.

#### 3-D Cell culture

The 3-D culture method was performed as described previously and modified [19]. Cells were maintained in complete KSFM medium containing 2% calf serum. When reached to 80% confluence, cells were harvested in serum-free trypsin-EDTA mixture and resuspended in DMEM/F12 medium supplemented with 2% horse serum, 0.5 μg/ml Hydrocortisone, 100 ng/ml Cholera toxin, 10 μg/ml insulin and 1x antibiotic-antimycotic mixture. The cells (2000/cm^2^) were mixed with 1.5 mg/ml collagen solution and seeded in eight-well chamber slides (Nalge Nunc, Naperville, IL) which pre-coated with 10 μl of 1.5 mg/ml type I collagen solution. After 30–60 min of solidation in incubator, the medium were added. The cells were grown in 5% CO_2_ at 37°C and were replenished with fresh medium every 3 days for a week followed by more frequent medium changes (every 2 days) thereafter.

### Trypan blue stain

After washed with PBS, the cells were detached with 0.5% trypsin/EDTA, then the cell suspension was mixed with 0.4% trypan blue (Merck, Germany) solution (9:1, final concentration 0.04%) and incubated for three minutes, the numbers of viable and nonviable cells were counted under microscope. Single cells were recovered from the extracellular matrix gel following digestion with dispase according to the manufacturer’s instruction (BD Biosciences). Cell viability was statistically calculated according to the following formula: living cell rate (%) = total number of live cells / (total number of live cells + dead cell number) × 100%.

### Ionizing radiation treatment

Ionizing radiation was administered with a PXi320 Irradiator (Precision X-ray Inc., USA). Different dose-rates, 86.76 cGy/min (160kV, 18mA), 20 cGy/min (104kV, 9mA), and 1.84 cGy/min (37kV, 9mA) were used. Priming doses (0.025 Gy to 0.1 Gy) were given at a dose rate of 1.84 cGy/min and challenging doses were given at dose rates of 86.76 cGy/min. Cells were treated with total absorption doses at room temperature 24 hours after seeding. Cells were harvested at different time points depending on different experiment design strategies.

### Western blot analysis

The treated or irradiated cells were washed with ice-cold PBS twice. The cell lysis buffer (25 mM Tris–HCl, pH 7.4, 25 mM NaCl, 0.5 mM EDTA, 1 mM sodium orthovanadate, 10 mM NaF, 25 mM β-glycerophosphate, 10 mM sodium pyrophosphate, 0.2 mM sodium molybdate, 10 mg/ml aprotinin, 2 mM phenylmethylsulfonyl fluoride, and 1% Triton X-100) was added to the cells and lysed by sonication for 60 s. Lysates were cleared by centrifugation for 15 min at 15,000g, and protein concentrations were determined using the BCA protein assay reagent (Pierce). Equal amounts of protein were separated by 10–12.5% SDS–PAGE, electrophoretically transferred to polyvinylidene difluoride membranes, and probed with primary antibodies and horseradish peroxidase-conjugated secondary antibodies (Promega), respectively. Immunoblots were developed by using the enhanced chemiluminescence (ECL) detection system (Amersham) according to the manufacturer’s protocol and autoradiography.

### Immunofluorescent staining for 3-D cultured cells

For immunofluorescence staining, the 3-D cultured cells were fixed in formalin for 30 minutes at room temperature followed by permeabilization with 0.5% Triton X-100, then two blocking steps were used for 3-D immunofluorescence staining. After blocking in staining buffer (130 mM NaCl, 7mM Na_2_HPO_4_, 3.5 mM NaH_2_PO_4_, 7.7 mM NaN_3_, o.1% BSA, 0.2% Triton X-100 and 0.05% Tween-20) supplemented by 10% normal goat serum for 1 hour at room temperature, the blocking buffer and goat anti-mouse F(ab’)2 fragment (Jachson ImmunoResearch #115–006–006) were added to cells on slides. Samples were incubated with primary antibodies. The Alexa Fluor 488-labeled goat anti-mouse secondary antibody was used (Molecular Probes, Eugene, OR) and the cells were counterstained with DAPI. Slides were mounted with fluorescent mounting medium (DAKO) and examined with fluorescence microscope. 50–100 cells were analyzed and the foci were counted by two researchers.

### Immunofluorescence staining in monolayer culture

Fibroblasts grown on glass cover slips were fixed with 2% formalin for 1 h and permeabilized with 0.2% Triton X-100 in PBS for 15 min on ice followed by blocking with 1% BSA in PBS. Immunofluorescent staining was performed by incubating with primary antibody for 1 h at 37°C and secondary antibody for 45 min at room temperature. The cover slips were mounted on a microscope slide with Fluomount. Cells were examined under fluorescent microscope. The numbers of positive cells were manually counted in 10–20 randomly chosen fields including 100–200 cells. Foci pictures were taken under the oil plane of microscope and the number of foci was determined per nucleus and the foci were counted by two researchers in each experiment.

### Statistical analysis

Data are expressed as mean ± SD. Statistical analyses of the above results were performed by a one-way analysis of variance (ANOVA) using the SPSS program (version 13.0) for windows (SPSS, Chicago, IL, USA). p < 0.05 was considered statistically significant.

## Results

### High dose radiation-induced DNA damage could be attenuated by pretreatment of low dose radiation in RMP-4 cells

**Fig 1:**
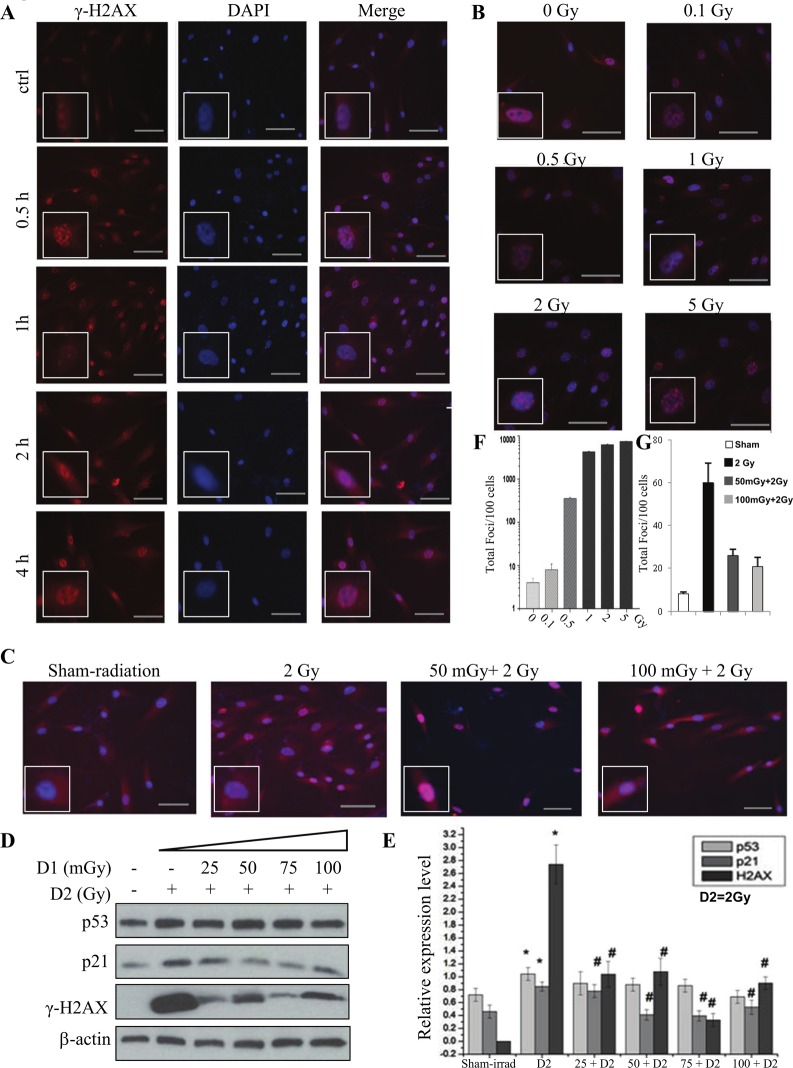
Radiation-induced AR in RMP-4 cells under 2-D condition. For priming dose (also named as D1), 25, 50, 75, 100 mGy of radiation were used. 2 Gy was used as challenging dose (D2), with a 6-hour interval. **(A)** The changes of γ-H2AX in RMP-4 cells by immunofluorescent staining after 2 Gy radiation in different time points. **(B)** The changes of γ-H2AX in RMP-4 cells by immunofluorescent staining at 1h after different doses of radiation. **(C)** The radiation-induced AR based on γ-H2AX changes. **(D)** The changes of γ-H2AX, p53, and p21 expression in radiation-induced AR by western blot. **(E)** The statistical analysis of γ-H2AX, p53 and p21 expression from Western blot. **(F)** The statistical analysis of γ-H2AX from immunofluorescent staining based on the foci numbers per cell after different doses of radiation. (G)The statistical analysis of γ-H2AX from immunofluorescent staining based on the foci numbers per cell after different dose rate of radiation.* *p*<0.05 vs control; # *p*<0.05 vs D2. The bar corresponds to 25 μm.

Ionizing radiation is one of the most important DNA damage inducers. The DSB is the mechanism. DSB maybe induced directly by ionizing energy or indirectly by secondary radicals [20]. Phosphorylation of H2AX at Ser 139 (γ-H2AX) is the most sensitive marker that can be used to examine the DNA damage and the subsequent repair of the DNA lesion [12, 13]. In these experiments, the γ-H2AX was used as the marker of DNA damage induced by radiation. The rationale was based on reports that H2AX is phosphorylated after exposure to X rays in a linear dose-dependent manner and is correlated with the presence of DSBs [14]. For priming dose (also named as D1), 25, 50, 75, 100 mGy of radiation were used, while 2 Gy was used for challenging dose (D2) with a 6-hour interval. Fig. 1A showed the time-course changes of γ-H2AX after 2 Gy radiation, the increase of γ-H2AX could be seen at 0.5 h after radiation and reached the maximum at 1 h, then decreased rapidly, but at 4 h after radiation the γ-H2AX was still visible.

To examine the dose-effect effects, different doses of radiation were used subsequently. We found the total numbers of foci increased in a dose-dependent manner (Fig. 1B and F). Since growing evidences supported the AR induced by low dose radiation, we designed different priming doses and a challenging dose (2 Gy) to see the effects on DNA damage. We found that the γ-H2AX positive foci significantly increased after 2 Gy of radiation and the priming low dose radiation could attenuate the increase of γ-H2AX positive foci induced by the challenging dose. These results were further verified by the γ-H2AX protein expression by western blot (Fig. 1C, D, E and G).

We further investigated the possible regulatory mechanisms by detecting the expression of p53 and p21. As shown in Fig. 1D and E, challenging dose of radiation increased the expression of total p53 and its downstream effect or p21 simultaneously. While the pretreatment of priming radiation (25mGy to 100mGy) decreased the expression of p21 induced by challenging radiation, total p53 expression did not change in RMP-4 cells.

### The low dose radiation induced adaptive response in IMR-90 cell line

**Fig 2:**
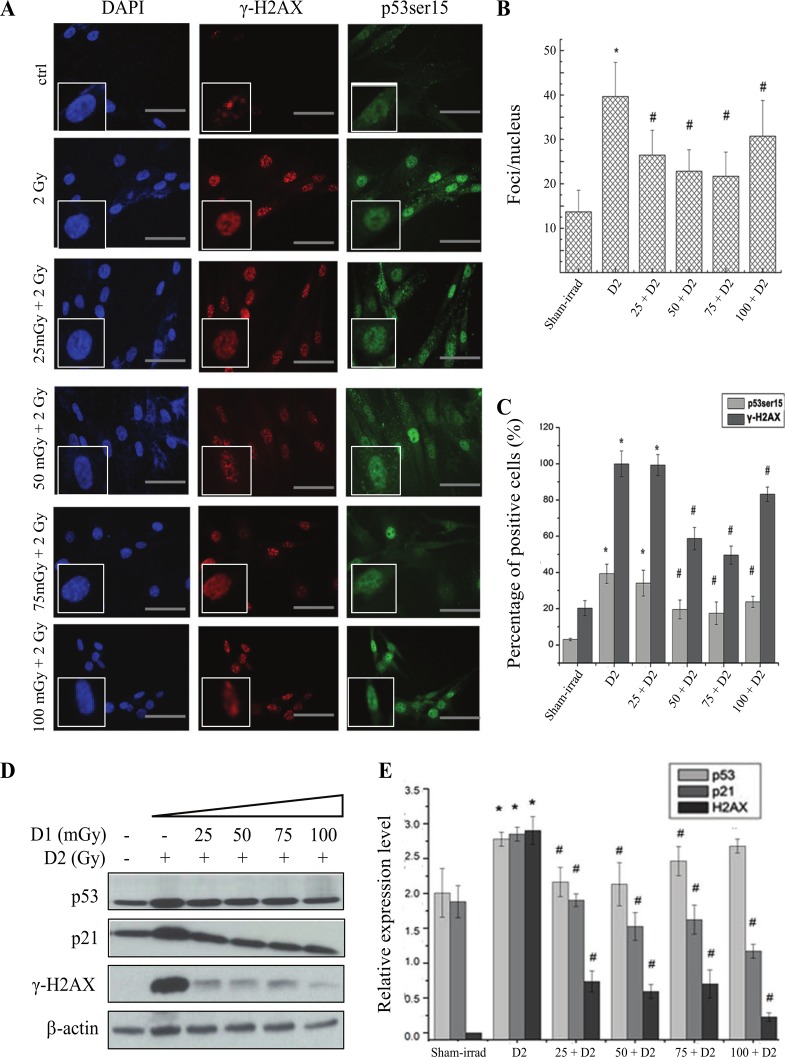
Radiation-induced AR in IMR-90 cells under 2-D condition. For priming dose (also named as D1), 25, 50, 75, 100 mGy of radiation were used. 2 Gy was used as challenging dose (D2), with a 6-hour interval. **(A)** The changes of γ-H2AX and p53 (ser-15) expression at 1h after D2 radiation in IMR-90 cells by immunofluorescent staining. **(B)** The statistical analysis of γ-H2AX from immunofluorescent staining based on the foci numbers per cell. **(C)** The statistical analysis of γ-H2AX and p53(ser-15) expression from immunofluorescent staining based on the percentage of positive cells. **(D)** The changes of γ-H2AX, p53 (ser-15) and p21 expression in radiation-induced AR by western blot. **(E)** The statistical analysis of γ-H2AX, p53 (ser-15) and p21 expression from Western blot. * *p*<0.05 vs control; # *p*<0.05 vs D2. The bar corresponds to 25 μm.

Two cell lines (IM-90 and MEF) were used to verify the low dose radiation-induced AR in fibroblasts. The immunofluorescent staining showed that the γ-H2AX foci significantly increased and the priming radiation could attenuate the increase of γ-H2AX (Fig. 2A and B). Since the priming radiation had no effect on the challenging radiation-induced changes of total p53 protein, the phosphorylation of p53 (Ser-15) was detected by both immunofluorescent staining and western blot in IM-90 and MEF cells. As shown in Fig. 2A, C, D and E, we found that challenging radiation increased the expression of p53 Ser-15 and p21, while the priming radiation, 25mGy to 100mGy (6 hrs interval), reversed it. Interestingly, the change of phosphorylated p53 was the same as that of γ-H2AX.

### The low dose radiation induced AR in MEF cell line

**Fig 3:**
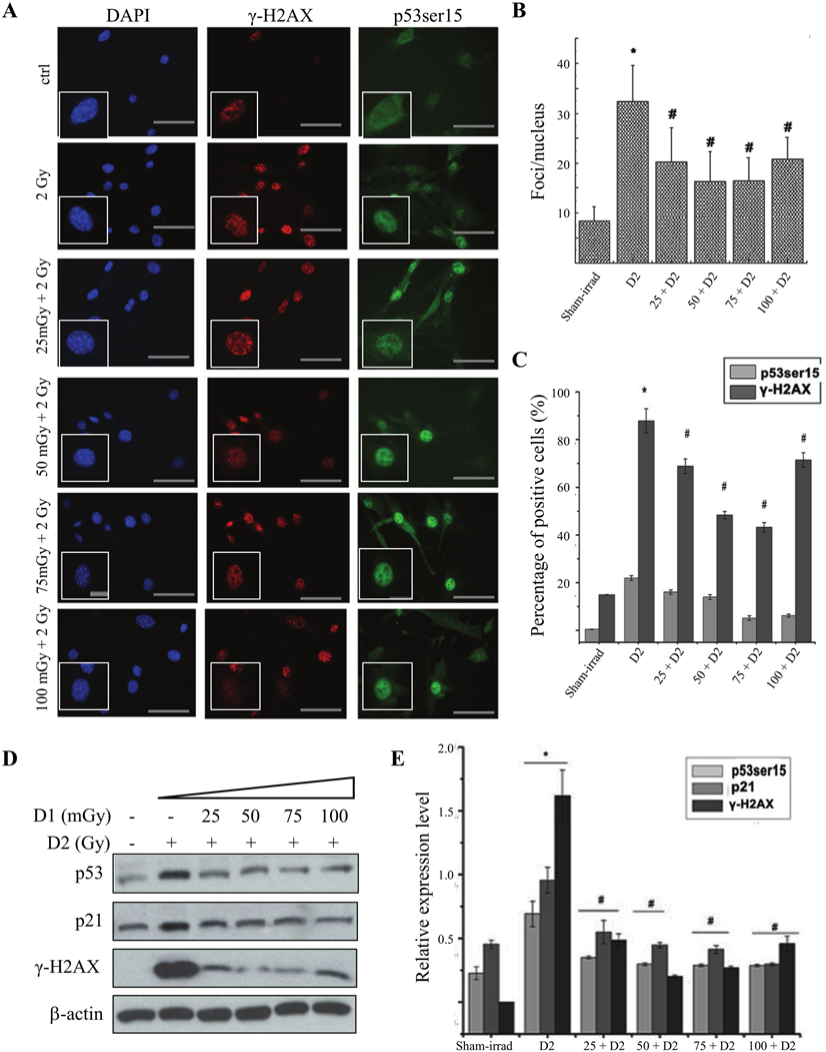
Radiation-induced AR in MEF cells under 2-D condition. For priming dose (also named as D1), 25, 50, 75, 100 mGy of radiation were used. 2 Gy was used as challenging dose (D2), with a 6-hour interval. **(A)** The changes of γ-H2AX and p53 (ser-15) expression after radiation in MEF cells by immunofluorescent staining. **(B)** The statistical analysis of γ-H2AX from immunofluorescent staining based on the foci numbers per cell. **(C)** The statistical analysis of γ-H2AX and p53 (ser-15) expression from immunofluorescent staining based on the percentage of positive cells. **(D)** The changes of γ-H2AX, p53 (ser-15) and p21 expression in radiation-induced AR by western blot. **(E)** The statistical analysis of γ-H2AX, p53 (ser-15) and p21 expression from Western blot. * *p*<0.05 vs control; # *p*<0.05 vs D2. The bar corresponds to 25 μm.

In MEF cells, 25, 50, 75, 100 mGy of radiation were used as priming dose, 2 Gy was used as challenging dose (D2), with a 6-hour interval. The foci of γ-H2AX, the expression of γ-H2AX and p53 Ser-15 were detected to observe the AR induced by low dose radiation. The immunofluorescent staining showed that the γ-H2AX and p53 Ser-15 significantly increased by D2 radiation. However, priming dose of radiation, 25mGy to 100mGy, reversed the D2-induced increased expression of γ-H2AX and p53 Ser-15 in a dose-dependent manner (Fig. 3A, B and C).

We also found that the expressions of γ-H2AX, p53 Ser-15 and p21 increased after D2 alone radiation in MEF cells. Different dose of D1 attenuated the increase of H2AX, p53 and p21(Fig. 3D and E). Our results suggested the potential roles of p53 and p21 in the regulation of DNA damage and repair.

### The comparison of priming radiation-induced AR between monolayer and 3D cell culture

**Fig 4:**
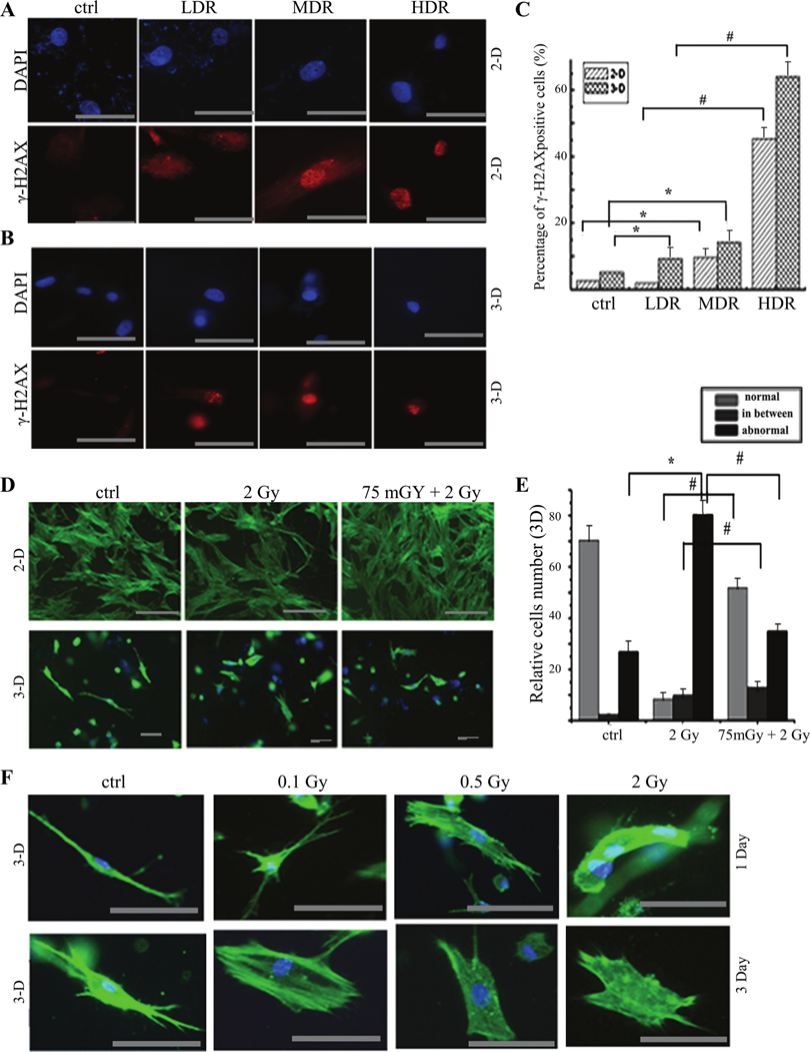
The comparison of morphological texture changes after radiation under 2-D and 3-D condition. RMP-4 cells were cultured under either 2-D or 3-D condition. The dose-effect analysis, dose rate-effect analysis and adaptive response were performed by immunofluorescent staining. **(A)** The changes of γ-H2AX expression after different dose rates of radiation (total 0.1 Gy) in RMP-4 cells under 2-D condition by immunofluorescent staining. **(B)** The changes of γ-H2AX expression after different dose rates of radiation (total 0.1 Gy) in RMP-4 cells under 3-D condition by immunofluorescent staining. **(C)** The statistical analysis of γ-H2AX from immunofluorescent staining after radiation under both 2-D and 3-D condition. **(D)** The AR induced by 75 mGy radiation under both 2-D and 3-D condition (pholloidin staining) based on the texture changes. **(E)** The statistical analysis of pholloidin staining after radiation under both 2-D and 3-D condition. Cells were divided into normal, abnormal and transit phase. **(F)** The changes of textures on 1 day or 3 days after different doses of radiation. * *p* <0.05 vs control, # *p* <0.05 vs 2 Gy. The bar corresponds to 25 μm.

To observe the difference of radiosensitivity between monolayer and 3-D condition, 3-D culture is used to mimic microenvironment and restore the cell shape and texture under the physiological condition. Low dose rate (LDR) 1.84 cGy/min, median dose rate (MDR) 20 cGy/min and high dose rate (HDR) 86.7 cGy/min (cumulative dose 0.5 Gy) were used in RMP-4 cells. γ-H2AX staining was used to monitor DNA damage. We found that the DNA damage significantly increased both under monolayer and 3-D condition as compared with the control group except LDR group under 2-D condition. Meanwhile, both under 2-D and 3-D culture condition, the DNA damage significantly increased at dose-dependent manner with different doses of radiation. The 3-D cultured cells were more sensitive to radiation-induced DNA damage (Fig. 4A, B and C).

To compare the difference of AR in cells cultured in monlayer and 3-D condition, the cells morphology were monitored by pholloidin staining. As shown in Fig. 4F, challenging dose of radiation made some RMP-4 cells lost their normal morphology and became shorten under 3-D condition, the pseudopodia disappeared and folded cells were observed. Some cells stayed in transit phase from the normal morphology to abnormal morphology. According to the morphology, we defined cells as normal, abnormal and transit in the following analysis. Then the difference of both morphological textures and AR were detected on 1 day after D2 radiation under 3-D and monolayer condition. The monolayer cells appeared stretch, dense and intertwined, some cells were shrinking but still kept veins and intertwining (Fig. 4D). However, 3-D cultured cells showed abnormal textures after 2 Gy radiation, and cells folded and the microspikes were observed. More cells with normal morphology were observed at priming 75 mGy at 6 hours before challenging irradiation, as compared with 2 Gy challenging irradiation only (Fig. 4D and E).

## Discussion

The biological effects of low dose radiation have been investigated for many years. These effects include low-dose hypersensitivity, increased radiation resistance, the adaptive response, the bystander effect and death-inducing factor activation [21–23]. The rationale of AR is the biological process that pretreatment of low dose radiation can make organism adapt to subsequent high dose radiation, and reduce the damage caused by high dose irradiation. The underlying molecular mechanisms of protective biological effects of low dose radiation mainly involve enhanced DNA repair, stimulated immune regulation and induced removal of damaged cells [24–27].

Low dose radiation shows various effects on organisms, depending on the low linear energy transfer (LET) of radiation, dose and dose rate, radiation modality. Low dose radiation could also trigger hypersensitivity. Acute damage of single units produced by a single low dose irradiation is greater than that of high doses, the dosage is usually in the range of 0.2 ~ 0.5 Gy. Radiation-induced bystander effect (RIBE) is the response of cells to their irradiated neighbors. The RIBE signals can attack the bystander cells and lead to biological effects including DNA damage, chromosomal aberration, gene mutation, malignant transformation and tumor formation [28–30].

The present studies verified the low dose radiation-induced AR under both monolayer and 3-D condition. We found the different changes in DNA damage and repair after low dose radiation. Although this 2-D culture has permitted numerous discoveries, almost all cells dramatically change function in such microenvironment, as compared with natural environment in vivo. Differentiated cells even lose their ability to express tissue-specific genes[31–34].

In our study, three non-dividing fibroblasts, RMP-4, IMR—90 and MFF were used and radiation-induced AR was observed under both 2-D and 3-D microenvironment. Based on the time-course and dose-effect experiments, the increase of γ-H2AX staining was seen at 0.5 h and 1 h after exposure to 2 Gy radiation. Under 2-D condition challenging dose of radiation caused DNA damage which was confirmed by phosphorylated H2AX foci, in a dose-dependent and time-dependent manner. However, priming low dose radiation attenuated the damage. Interestingly, cells were more sensitive to radiation under 3-D condition. Different dose rates of radiation induced more DNA damage under 3-D than that under 2-D condition (Fig. 4A, B and C). Dose rate effect of radiation has been long admitted[35]. The H2AX expression increased in dose dependent manner (Fig. 4A, B and C), which is consistent with the traditional radiobiological theory. Considering the different radiosensitivity between monolayer and 3-D culture, the fate of cells after radiation will be determined by the microenvironment, the suboptimal condition will slow down the proliferation of cells and contribute to the potential lethal damage repair. According to the Bergonie Tribondeau theory, the radiosensitivity is proportional to the proliferation and division, and is inversely proportional to differentiation. The above mentioned results and a lower amount of DNA damage manifested better survival after AR (data not shown). These data suggested the enhanced radiosensitivity under 3-D as compared with that under 2-D condition.

Pholloidin staining was also used to detect the texture in this study. As shown in Fig. 4D, E and F, there were three types of cells: normal, abnormal and transit phase (atypical cells). Under 2-D culture condition there was little difference between 2 Gy and 75mGy+2 Gy groups based on morphological texture (Fig. 4D). However, the morphology is significantly different between control, 2 Gy and 75mGy+2Gy groups (Fig. 4D and E) under 3-D culture condition. With challenging radiation of 0.1, 0.5 and 2 Gy, folded cells and the microspikes were observed. The shrinked cells, microfilament and pseudopod disappeared on 1 and 3 days after radiation (Fig. 4F). Under 3-D condition the challenging dose of 2 Gy increased the percentage of abnormal cells (286% of sham-radiation group). Priming dose of 75 mGy attenuated this change, and the percentage of abnormal cells decreased to almost 132% of sham-radiation group (Fig. 4D and E). Although AR can be induced under 2-D condition, the DNA damage by D2 and the attenuation by D1 were less than that under 3-D condition. The changes of texture indicated that AR was induced in fibroblasts.

The phosphatidylinositol-3-kinase-protein kinase B/Akt (PI3K-PKB/Akt) signal transduction pathway associates with tumorigenesis and resistance to radiotherapy. Ras signaling plays important roles in various cellular processes, including proliferation, differentiation and apoptosis. Lee et al found that the response of protein kinase C (PKC) isozymes is different between normal and neoplastic mouse epidermal cells after primed with a low dose of gamma-rays followed by high dose of gamma-rays[36]. Their study indicated that responsiveness of PKC affects this adaptive response. Prasad et al found low dose radiation induced different amounts of the proto-oncogenes expression, such as c-fos, c-jun, c-myc and c-Ha-ras [37]. Studies also indicated that low dose radiation stimulates cell proliferation by transit activation of PI3K/Akt pathway. The p53 protein plays a key role in the adaptive response, especially in regulating radiation-induced DSBs [38]. As an important transcriptional factor, p53 also participates in the process of DNA damage and repair, the senescence, the cell death. Our study showed that D2 radiation increased the expression of p53, γ-H2AX and p21. Pre-exposure to 25, 50, 75 and 100 mGy of D1 radiation induced an AR to subsequent D2-induced expression of γ-H2AX. It was more effective at 50 and 75 mGy. However, no changed of total p53 protein was observed at priming dose of D1 (Fig. 1D). Further study indicated that D2 increased p53 Ser-15, γ-H2AX and the downstream effect or p21, suggesting that phosphorylated p53 might participate in the AR. The previous study indicated that cells response to high doses of radiation by activation of ERK and JNK kinases and WIP phosphatase. Further study need to be done to examine the function of p53 on low dose radiation-induced AR and the signaling pathway involved the cell response to high doses of radiation.

In a summary, the low dose radiation could induce AR both under 2-D and 3-D condition. The fibroblasts are more resistant to DNA damage under 2-D culture than that under 3-D culture condition probably due to the existence of potential lethal damage repair. P53 and p21 might associate with the regulation of radiation-induced AR. Further studies need to be done to examine the exact mechanisms.
